# Data of programmed death-ligand 1 expression and VEGF: Nivolumab, bevacizumab and paclitaxel For HER2-negative metastatic breast cancer

**DOI:** 10.1016/j.dib.2022.108558

**Published:** 2022-09-01

**Authors:** Yukinori Ozaki, Junji Tsurutani, Toru Mukohara, Tsutomu Iwasa, Masato Takahashi, Yuko Tanabe, Hidetaka Kawabata, Norikazu Masuda, Manabu Futamura, Hironobu Minami, Koji Matsumoto, Kenichi Yoshimura, Shigehisa Kitano, Toshimi Takano

**Affiliations:** a Department of Medical Oncology, Toranomon Hospital, Tokyo, Japan; b Department of Breast Medical Oncology, The Cancer Institute Hospital of Japanese Foundation for Cancer Research, Tokyo, Japan; c Advanced Cancer Translational Research Institute, Showa University, Tokyo, Japan; d Department of Medical Oncology, National Cancer Center Hospital East, Kashiwa, Japan; e Department of Medical Oncology, Faculty of Medicine, Kindai University, Osaka, Japan; f Department of Breast Surgery, National Hospital Organization Hokkaido Cancer Center, Hokkaido, Japan; g Department of Breast and Endocrine Surgery, Toranomon Hospital, Tokyo, Japan; h Department of Surgery, Breast Oncology, National Hospital Organization Osaka National Hospital, Osaka, Japan; i Department of Breast and Endocrine Surgery, Graduate School of Medicine, Nagoya University, Nagoya, Japan; j Breast Surgery, Gifu University Hospital, Gifu, Japan; k Medical Oncology/Hematology, Internal Medicine, School of Medicine, Kobe University, Hyogo, Japan; l Department of Medical Oncology, Hyogo Cancer Center, Hyogo, Japan; m Center for Integrated Medical Research, Hiroshima University Hospital, Hiroshima University, Hiroshima, Japan; n Department of Experimental Therapeutics, National Cancer Center Hospital, Tokyo, Japan; o Department of Advanced Medical Development, The Cancer Institute Hospital of Japanese Foundation for Cancer Research, Tokyo, Japan.

**Keywords:** Nivolumab, Bevacizumab, Immunotherapy, PD-L1, VEGF, Metastatic breast cancer

## Abstract

The purpose was to explore potential biomarkers of the efficacy and toxicity of triple therapy of nivolumab, bevacizumab and paclitaxel in patients with HER2-negative metastatic breast cancer (MBC). Tumor tissues before treatment and blood samples at pretreatment, during and after treatment were collected. The serum samples were used to measure the concentrations of cytokines. Progression-free survival (PFS), overall survival (OS), and response were analyzed in association with the biomarker data using the Kaplan–Meier method and log-rank tests. Fifty patients were included in the biomarker analysis. Programmed death-ligand 1 (PD-L1) expression on tumor cells and immune cells were evaluated in tumor tissue samples using a Dako 28-8 immunohistochemistry assay and using a VENTANA SP142 immunohistochemistry assay. PD-L1 positive rates using anti-PD-L1 antibodies 28-8 (Combined positive score [CPS] ≥1) and SP142 (Immune cells [IC] ≥1) were 15% and 17%, respectively. The PFS and OS were not significantly different in the subgroups by PD-L1 expression. The median pretreatment vascular endothelial growth factor (VEGF)-A concentration was 116.1 pg/ml (range 0–740.23 pg/ml) on day 1 and decreased to <37 pg/ml on day 8 of cycle 1 in all patients. Subtypes (hormone receptor-positive HER2-negative or triple negative breast cancer), stage (recurrent or *de novo* stage IV) and liver metastasis (yes or no) were not significantly different between patients in VEGF-A high and VEGF-A low groups. PFS in the VEGF-A high group was similar to that in the VEGF-A low group.

**Specifications Table**

**Table:** 

Subject	Health and medical sciences: Oncology
Specific subject area	Combination immunotherapy with an immune checkpoint inhibitor and anti-VEGF antibody in metastatic breast cancer
Type of data	Tables and Figures
How the data were acquired	The investigators collected tumor tissues (archival tissue or biopsy of metastatic site) and blood samples from patients who received nivolumab, bevacizumab and paclitaxel. Peripheral blood mononuclear cells and serum samples were collected at pretreatment, cycle 1 day 8, cycle 2 day 1, cycle 3 day 1, and cycle 8 day 1, or at the time of nivolumab discontinuation, and at the time of disease progression. The investigators could collect samples when an immune-related adverse event occurred.
Data format	Raw and Analyzed
Description of data collection	The main eligibility criteria were women aged ≥20 years with histologically confirmed invasive, metastatic, or inoperable HER2-negative breast cancer. Key exclusion criteria were prior treatment with immune checkpoint inhibitors or treatment with systemic corticosteroids at a prednisolone equivalent dose of >10 mg/day.
Data source location	Department of Breast Medical Oncology The Cancer Institute Hospital of Japanese Foundation for Cancer Research 3-8-31 Ariake, Koto-ku, Tokyo 135-8550, Japan Tel: +81 3 3520 0111
Data accessibility	Repository name: Mendeley Data Title of dataset: Data of Programmed death-ligand 1 expression and VEGF: Nivolumab Plus Bevacizumab, Paclitaxel For HER2-Negative Metastatic Breast Cancer (WJOG9917BTR) Data identification number: http://dx.doi.org/10.17632/kfwz4s83zp.4
Related research article	Y. Ozaki, J. Tsurutani, T. Mukohara, T. Iwasa, M. Takahashi, Y. Tanabe, H. Kawabata, N. Masuda, M. Futamura, H. Minami, K. Matsumoto, K. Yoshimura, S. Kitano, T. Takano, Safety and efficacy of nivolumab plus bevacizumab, paclitaxel for HER2-negative metastatic breast cancer: Primary results and biomarker data from a phase 2 trial (WJOG9917B), Eur J Cancer 171 (2022) 193-202. https://doi.org/10.1016/j.ejca.2022.05.014.

## Value of the Data

• Data to evaluate the association of PD-L1 expression and serum VEGF levels with the efficacy of the combination therapy of immune checkpoint inhibitor and anti-VEGF agent in breast cancer.
• These data contribute to the development of treatment for patients receiving first-line chemotherapy for HER2-negative metastatic breast cancer.
• Understanding dynamic changes of serum VEGF in patients who have received an immune checkpoint inhibitor and anti-VEGF agent can lead to further development of the combination treatment.

## 1 Data Description

### 1.1 NEWBEAT Protocol (Version 1.0)

In patients with advanced metastatic or incurable recurrent breast cancer with HER2-negative regardless of PD-L1 expression, we examined the effectiveness and safety of nivolumab, bevacizumab, and paclitaxel as the first-line triple treatment in NEWBEAT (WJOG9917B). The design of the experiment was a joint effort between eight West Japan Oncology Group institutions. Institutional review boards at each of the participating institutions gave their approval, which was carried out in accordance with Good Clinical Practice and the Declaration of Helsinki. After ensuring that the patients understood the study completely, the researchers received signed consent from the participants. (WJOG9917B: UMIN000030242) (Table 1).

**Table 1:** PD-L1 positive rate using anti-PD-L1 antibodies 28-8 and SP142 in all patients and according to breast cancer type. The PD-L1 positivity rate using anti-PD-L1 antibodies 28-8 (CPS ≥ 1) was 15% in all patients. A lower positivity rate was observed in hormone receptor (HR)-positive HER2-negative subtype (6%) compared with triple negative breast cancer (TNBC) (36%). Using TPS ≥ 1, the PD-L1 positivity rate was 13% in all patients, 3% in HR-positive HER2-negative subtype and 36% in TNBC. The analysis using IC ≥ 1 showed the PD-L1 positivity rate was 20% in all patients, 6% in HR-positive HER2-negative subtype and 50% in TNBC. The PD-L1 positivity rate using anti-PD-L1 antibodies SP142 (IC ≥ 1) was 17% in all patients. A lower positivity rate was observed in HR-positive HER2-negative subtype (6%) compared to TNBC (43%). The raw data included patient number, subtype, TPS, CPS, IC with 28-8 and SP142.

	TPS* ≥1% Antibody 28-8	CPS† ≥1 Antibody 28-8	IC‡ ≥1 Antibody 28-8	IC‡ ≥1 Antibody SP142

	*n* (%) of patients			
All patients (*N* = 46)	6 (13)	7 (15)	9 (20)	8 (17)
HR+ HER2− (*N* = 32)	1 (3)	2 (6)	2 (6)	2 (6)
TNBC (*N* = 14)	5 (36)	5 (36)	7 (50)	6 (43)

⁎ TPS is the tumor positive score, and was calculated as the number of PD-L1-positive tumor cells/number of tumor cells × 100 (%).

† CPS is the combined positive score, classified as CPS ≥ 1 or CPS < 1, and is evaluated as the percentage of PD-L1-positive cells (tumor cells, lymphocytes, or macrophages)/number of tumor cells.

‡ IC is the percentage of immune cells in the tumor area and is classified as IC0 (<1%), IC1 (≥1% to <5%), IC2 (≥5% to <10%), or IC3 (≥10%). Abbreviations: PD-L1, programmed cell death ligand-1; HR, hormone receptor; HER2, human epidermal growth factor receptor 2; TNBC, triple-negative breast cancer.

**Table 2:** Characteristics of patients in the VEGF-A high and VEGF-A low subgroups. Serum VEGF-A concentrations were measured in 50 patients. The median concentration of serum VEGF-A was 116.1 pg/ml (range 0–740.23 pg/ml) before treatment. The patients were divided into those with VEGF concentrations above (VEGF-A high) or below (VEGF-A low) the median concentration and their baseline characteristics are presented in Table 2. The median age of patients was 49 in the VEGF-A high group and 46 in the VEGF-A low group. HR-positive HER2-negative subtype accounted for 64% and 68% in the VEGF-A high and VEGF-A low group, respectively. Recurrent disease was 80% in the VEGF-A high group compared with 64% in the VEGF-A low group. Liver metastasis was observed in 40% and 32% of the VEGF-A high and VEGF-A low group, respectively. Neoadjuvant or adjuvant treatment was performed in 84% and 64% of the VEGF-A high and VEGF-A low group, respectively. Treatment including taxane regimen was performed in 64% and 40% of the VEGF-A high and VEGF-A low group, respectively. The raw data included patient number, age, subtype, stage (*de novo* stage IV or recurrent disease), liver metastasis, neoadjuvant or adjuvant treatment, prior taxane and pre-treatment serum VEGF-A concentration (pg/ml).

	VEGF-A_high_ (VEGF-A ≥116 pg/ml) (*N* = 25)	VEGF-A_low_ (VEGF-A <116 pg/ml) (*N* = 25)

Age		
Median (range), years	49 (40–76)	46 (31–74)
Subtype, n (%)		
HR+ HER2−TNBC	16 (64)	17 (68)
	9 (36)	8 (32)
Stage, n (%)		
*De novo* stage 4	5 (20)	9 (36)
Recurrent breast cancer	20 (80)	16 (64)
Liver metastasis		
Yes	10 (40)	8 (32)
No	15 (60)	17 (68)
Neo/adjuvant treatment, n (%)	21 (84)	16 (64)
Prior taxane regimen (neo/adjuvant), n (%)	16 (64)	10 (40)

Abbreviations: VEGF-A, vascular endothelial growth factor-A; HR, hormone receptor; HER2, human epidermal growth factor receptor 2; TNBC, triple-negative breast cancer.

**Fig. 1:**
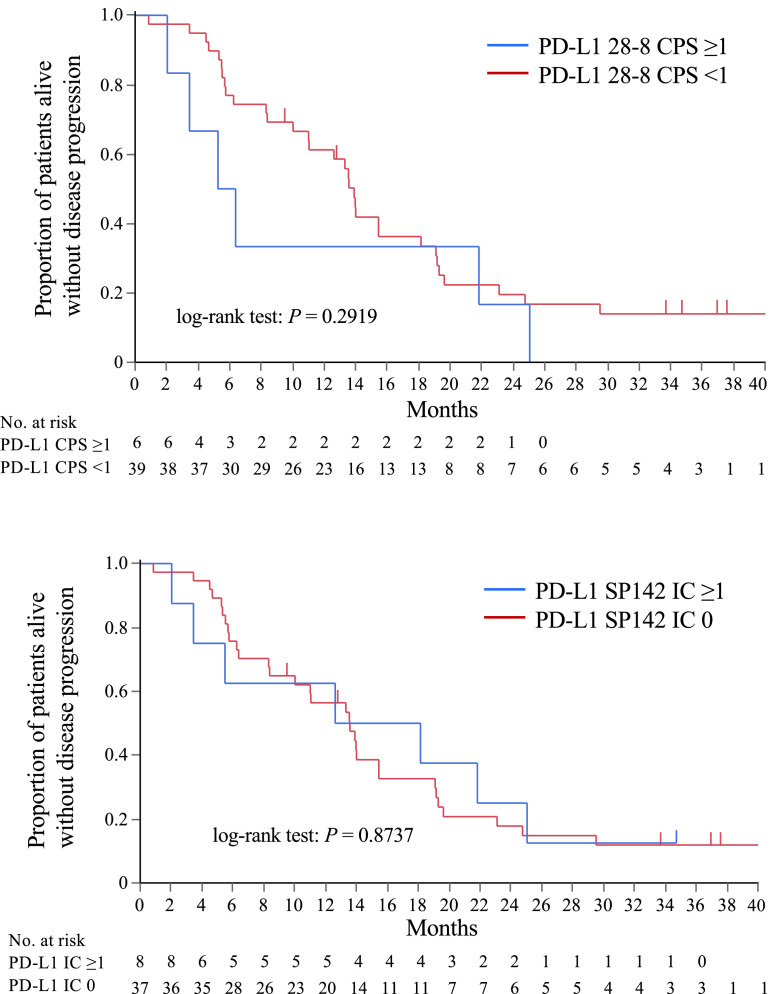
Progression-free survival according to biomarker expression. Fig. 1 shows the PFS data according to PD-L1 expression using 28-8 (CPS ≥1 or <1) and SP142 (IC ≥1 or <1). The log-rank test showed p value was 0.2919 using 28-8, and 0.8737 using SP142, respectively. The raw data included PFS rate, survival standard error, 95% confidence interval, number of event, number of censored and number at risk using 28-8 and SP142. Abbreviations: CPS, combined positive score; IC, immune cell; PD-L1, programmed cell death ligand-1.

**Fig. 2:**
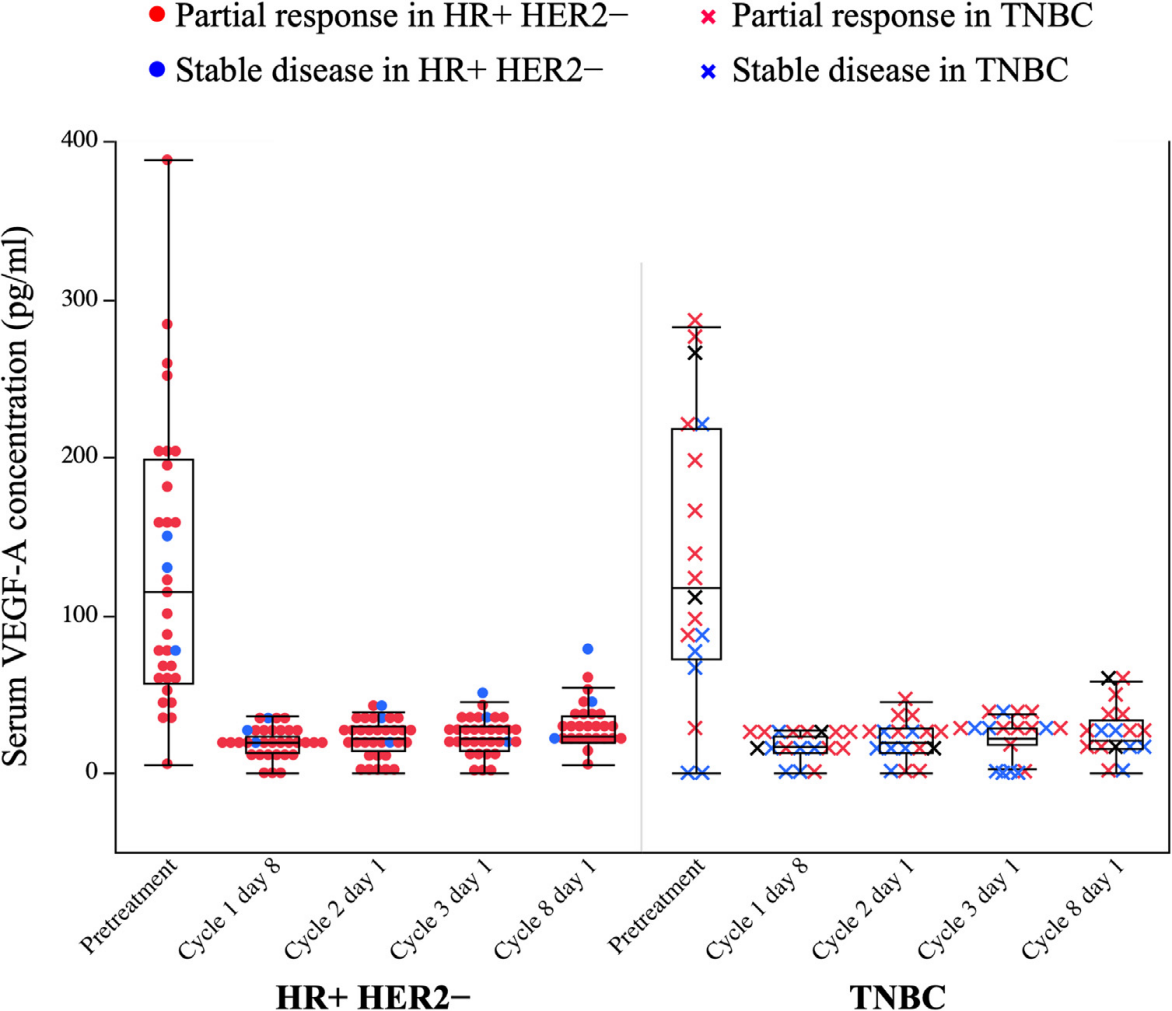
Serum VEGF-A concentrations and best response according to tumor subtype. Fig. 2 shows the serum VEGF-A concentrations in pretreatment, cycle 1 day 8, cycle 2 day 1, cycle 3 day 1, and cycle 8 day 1, or at the time of nivolumab discontinuation according to tumor subtypes. The serum VEGF-A concentrations decreased to <37 pg/ml on day 8 of cycle 1 in all patients. Red: partial response, Blue: stable disease, circle: HR+ HER2-, x: TNBC. Box plot shows 95% confidential interval. The raw data included patient number, time point, best response, stage (*de novo* stage IV or recurrent disease), subtype, PFS, PFS event, OS, OS event, serum VEGF-A concentration (pg/ml). Abbreviations: VEGF-A, vascular endothelial growth factor-A; HR, hormone receptor; HER2, human epidermal growth factor receptor 2; TNBC, triple negative breast cancer.

**Fig. 3:**
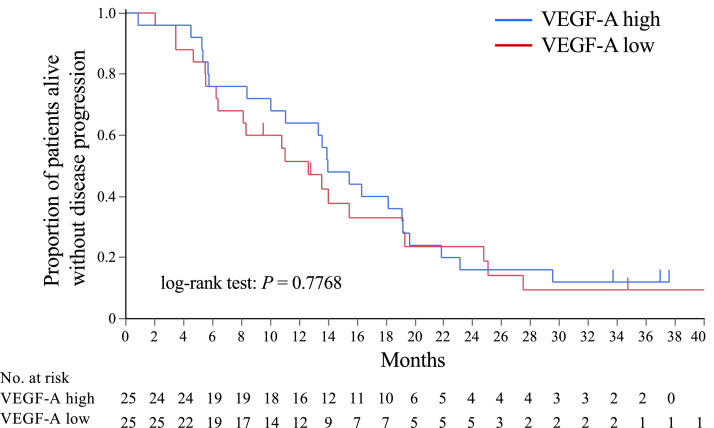
Progression-free survival according to VEGF-A expression. Fig. 3 shows the PFS data according to pretreatment VEGF-A level. The log-rank test showed p value was 0.7768. The raw data included PFS rate, survival standard error, 95% confidence interval, number of event, number of censored and number at risk. Abbreviations: VEGF-A, vascular endothelial growth factor-A.

## 2 Experimental Design, Materials and Methods

The IMpassion130 and KEYNOTE355 demonstrated the efficacy and safety of atezolizumab, an anti-PD-L1 antibody, and pembrolizumab, an anti-PD-1 antibody, combined with chemotherapy for patients with PD-L1–positive advanced TNBC [1,2]. Nivolumab is a human monoclonal anti-PD-1 antibody (IgG4) that inhibits PD-1–PD-L1 binding, enhances antigen-specific T-cell activity, and increases immune responses to cancer [3]. VEGF-A plays immunosuppressive effects, is a key regulator of angiogenesis, and is abundantly expressed in many cancers. Myeloid-derived suppressor cells, immature dendritic cells, regulatory T-cells, and tumor-associated macrophages are all induced by VEGF-A [4]. Bevacizumab affects the tumor immune system by reducing regulatory T cells and bone marrow-derived suppressor cells within the tumor microenvironment, downregulating immune-exhausted molecules, weaning VEGF-related immunosuppression, promoting dendritic cell maturation and T-cell priming, normalizing the tumor vasculature, and increasing T-cell infiltration into the tumor [5,6]. Coadministration of a VEGF-A inhibitor and an anti-PD-1 antibody have shown clinical benefit in non-small lung cancer (NSCLC), renal cell carcinoma, and hepatocellular carcinoma [7], [8], [9], [10]. We evaluated the efficacy and safety of first-line triple therapy comprising nivolumab, bevacizumab, and paclitaxel in patients with HER2-negative advanced metastatic or inoperable recurrent breast cancer [11]. The purpose of WJOG9917BTR is to explore potential biomarkers of the efficacy and toxicity of triple therapy in patients with HER2-negative MBC. The investigators collected tumor tissues (archival tissue or biopsy of metastatic site) and blood samples from consenting patients. Peripheral blood mononuclear cells and serum samples were collected at pretreatment, cycle 1 day 8, cycle 2 day 1, cycle 3 day 1, and cycle 8 day 1, or at the time of nivolumab discontinuation, and at the time of disease progression. Tumor tissue samples were used to evaluate PD-L1 expression on tumor cells and immune cells using a Dako 28-8 immunohistochemistry assay and on immune cells using a VENTANA SP142 immunohistochemistry assay (Table 1). Peripheral blood mononuclear cells were used to analyze the immune cell profile and comprehensive gene analysis by flow cytometry and the nucleic acid digital counting method. The serum samples were used to measure the concentrations of cytokines, chemokines, and other surrogate proteins. Progression-free survival, overall survival, and response were analyzed in association with the biomarker data using the Kaplan–Meier method, log-rank tests, Cox proportional hazards model, and logistic model regression analysis as appropriate.

## Ethics Statements

These data were collected according to Good Clinical Practice and the Declaration of Helsinki, and approved by institutional review boards at all participating sites. The investigators obtained a written consent from the patient. (WJOG9917BTR: UMIN000029590).

## CRediT authorship contribution statement

**Yukinori Ozaki:** Conceptualization, Data curation, Formal analysis, Funding acquisition, Methodology, Visualization. **Junji Tsurutani:** Conceptualization, Funding acquisition, Supervision, Visualization. **Toru Mukohara:** Formal analysis, Visualization. **Tsutomu Iwasa:** Formal analysis, Visualization. **Masato Takahashi:** Formal analysis, Visualization. **Yuko Tanabe:** Formal analysis, Visualization. **Hidetaka Kawabata:** Formal analysis, Visualization. **Norikazu Masuda:** Formal analysis, Visualization. **Manabu Futamura:** Formal analysis, Visualization. **Hironobu Minami:** Formal analysis, Visualization. **Koji Matsumoto:** Formal analysis, Visualization. **Kenichi Yoshimura:** Data curation, Formal analysis, Validation, Visualization. **Shigehisa Kitano:** Conceptualization, Formal analysis, Supervision, Visualization. **Toshimi Takano:** Conceptualization, Funding acquisition, Methodology, Supervision, Visualization.

## Declaration of Competing Interest

The authors declare the following financial interests/personal relationships which may be considered as potential competing interests.

## Data Availability

Data of Programmed death-ligand 1 expression and VEGF: Nivolumab Plus Bevacizumab, Paclitaxel For HER2-Negative Metastatic Breast Cancer (WJOG9917BTR) (Original data) (Mendeley Data)

## References

[1] Schmid P., Adams S., Rugo H.S., Schneeweiss A., Barrios C.H., Iwata H., Dieras V., Hegg R., Im S.A., Shaw Wright G., Henschel V., Molinero L., Chui S.Y., Funke R., Husain A., Winer E.P., Loi S., Emens L.A., I.M.T. Investigators (2018). Atezolizumab and nab-paclitaxel in advanced triple-negative breast cancer. N. Engl. J. Med..

[2] Cortes J., Cescon D.W., Rugo H.S., Nowecki Z., Im S.A., Yusof M.M., Gallardo C., Lipatov O., Barrios C.H., Holgado E., Iwata H., Masuda N., Otero M.T., Gokmen E., Loi S., Guo Z., Zhao J., Aktan G., Karantza V., Schmid P., K.-. Investigators (2020). Pembrolizumab plus chemotherapy versus placebo plus chemotherapy for previously untreated locally recurrent inoperable or metastatic triple-negative breast cancer (KEYNOTE-355): a randomised, placebo-controlled, double-blind, phase 3 clinical trial. Lancet.

[3] Scott L.J. (2015). Nivolumab: a review in advanced melanoma. Drugs.

[4] Voron T., Marcheteau E., Pernot S., Colussi O., Tartour E., Taieb J., Terme M. (2014). Control of the immune response by pro-angiogenic factors. Front. Oncol..

[5] Huang Y., Goel S., Duda D.G., Fukumura D., Jain R.K. (2013). Vascular normalization as an emerging strategy to enhance cancer immunotherapy. Cancer Res..

[6] Georganaki M., van Hooren L., Dimberg A. (2018). Vascular targeting to increase the efficiency of immune checkpoint blockade in cancer. Front. Immunol..

[7] Wallin J.J., Bendell J.C., Funke R., Sznol M., Korski K., Jones S., Hernandez G., Mier J., He X., Hodi F.S., Denker M., Leveque V., Canamero M., Babitski G., Koeppen H., Ziai J., Sharma N., Gaire F., Chen D.S., Waterkamp D., Hegde P.S., McDermott D.F. (2016). Atezolizumab in combination with bevacizumab enhances antigen-specific T-cell migration in metastatic renal cell carcinoma. Nat. Commun..

[8] McDermott D.F., Huseni M.A., Atkins M.B., Motzer R.J., Rini B.I., Escudier B., Fong L., Joseph R.W., Pal S.K., Reeves J.A., Sznol M., Hainsworth J., Rathmell W.K., Stadler W.M., Hutson T., Gore M.E., Ravaud A., Bracarda S., Suarez C., Danielli R., Gruenwald V., Choueiri T.K., Nickles D., Jhunjhunwala S., Piault-Louis E., Thobhani A., Qiu J., Chen D.S., Hegde P.S., Schiff C., Fine G.D., Powles T. (2018). Clinical activity and molecular correlates of response to atezolizumab alone or in combination with bevacizumab versus sunitinib in renal cell carcinoma. Nat. Med..

[9] Socinski M.A., Jotte R.M., Cappuzzo F., Orlandi F., Stroyakovskiy D., Nogami N., Rodriguez-Abreu D., Moro-Sibilot D., Thomas C.A., Barlesi F., Finley G., Kelsch C., Lee A., Coleman S., Deng Y., Shen Y., Kowanetz M., Lopez-Chavez A., Sandler A., Reck M., I.M.S. Group (2018). Atezolizumab for first-line treatment of metastatic nonsquamous NSCLC. N. Engl. J. Med..

[10] Finn R.S., Qin S., Ikeda M., Galle P.R., Ducreux M., Kim T.Y., Kudo M., Breder V., Merle P., Kaseb A.O., Li D., Verret W., Xu D.Z., Hernandez S., Liu J., Huang C., Mulla S., Wang Y., Lim H.Y., Zhu A.X., Cheng A.L., I.M. Investigators (2020). Atezolizumab plus bevacizumab in unresectable hepatocellular carcinoma. N. Engl. J. Med..

[11] Ozaki Y., Tsurutani J., Mukohara T., Iwasa T., Takahashi M., Tanabe Y., Kawabata H., Masuda N., Futamura M., Minami H., Matsumoto K., Yoshimura K., Kitano S., Takano T. (2022). Safety and efficacy of nivolumab plus bevacizumab, paclitaxel for HER2-negative metastatic breast cancer: Primary results and biomarker data from a phase 2 trial (WJOG9917B). Eur. J. Cancer.

